# Randomized, Double-Blind, Crossover Trial Comparing Low-Glycemic Index Functional and Conventional Wholegrain Carbohydrates on Glycolipid Metabolism and Vascular Stress Markers in Adults with Suboptimal Triglyceridemia: The GLOW Study

**DOI:** 10.3390/jcm15051745

**Published:** 2026-02-25

**Authors:** Marina Giovannini, Federica Fogacci, Cristina Scollo, Valentina Di Micoli, Elisa Grandi, Arrigo F. G. Cicero

**Affiliations:** 1 Hypertension and Cardiovascular Risk Factors Research Centre, Medical and Surgical Sciences Department, Alma Mater Studiorum University of Bologna, 40100 Bologna, Italy; marina.giovannini3@unibo.it (M.G.); federica.fogacci@studio.unibo.it (F.F.); valentina.dimicoli2@unibo.it (V.D.M.); elisa.grandi@unibo.it (E.G.); 2 Department of Medical Pharmacology, Medical Faculty, Ataturk University, Erzurum 25240, Turkey; 3 Italian Society of Nutraceuticals (SINut), 40100 Bologna, Italy; 4 Cardiovascular Medicine Unit, IRCCS Azienda Ospedaliero-Universitaria di Bologna, 40100 Bologna, Italy; cristina.scollo@aosp.bo.it

**Keywords:** low-glycemic index, triglyceride–glucose index, cardiometabolic risk, functional carbohydrates, endothelial function

## Abstract

Mild fasting hypertriglyceridemia is often accompanied by early insulin resistance and atherogenic dyslipidemia, making it an attractive target for pragmatic dietary prevention. This trial aims to determine whether substituting common cereal-based staples with functional low-glycemic index (low-GI) products improves the triglyceride–glucose (TyG) index in adults with fasting triglycerides >150 mg/dL. The GLOW study is an exploratory, randomized, double-blind, single-center crossover trial. Adults aged ≥18 years with fasting triglycerides >150 mg/dL and body mass index ≤30 kg/m^2^ will be enrolled. Participants will follow a stabilized Mediterranean-style diet and will complete two 28-day intervention periods in random sequence: (i) functional low-GI Altograno^®^ pasta, pizza base and flatbread; and (ii) conventional standard wholegrain products. Intervention periods will be separated by a 28-day washout. Study foods will be consumed as fixed daily substitutions of usual staple servings (one bread portion and one pasta or pizza portion). The primary endpoint is the between-intervention difference in TyG response over each period, defined as the period-specific change from the corresponding period baseline to the end-of-period assessment. The primary analysis will compare end-of-period TyG between interventions while adjusting for the period-specific baseline value. Secondary endpoints include fasting triglycerides and glucose, atherogenic lipoproteins (non–high-density lipoprotein cholesterol and apolipoprotein B), inflammation (high-sensitivity C-reactive protein), endothelial reactivity assessed with the Endocheck^®^/Vicorder^®^ system, and food acceptability. Safety endpoints include adverse event recording. Treatment effects will be estimated using linear mixed-effects models accounting for treatment, period and sequence, with prespecified carryover sensitivity analyses. A total of 40 participants will be recruited to generate feasibility data and effect size estimates. This protocol will provide crossover evidence on whether pragmatic, product-level low-GI staple substitution improves TyG and related cardiometabolic and vascular biomarkers in adults with suboptimal triglyceridemia, informing larger trials. Trial registration: ClinicalTrials.gov NCT07198789.

## 1. Introduction

Dietary factors play a major role in shaping the global burden of cardiovascular disease (CVD) and represent a key target for prevention strategies worldwide [1]. In analyses derived from the Global Burden of Disease (GBD) 2019 across 204 countries and territories (1990–2019), approximately 40% of CVD mortality and disability-adjusted life years (DALYs) were attributable to dietary risk factors, with high sodium intake, low whole-grain intake, and low legume intake consistently ranking among the most influential contributors globally [1]. These observations underscore the relevance of dietary strategies focused on improving carbohydrate quality and staple-food choices as pragmatic levers to reduce cardiometabolic risk at scale [1].

In parallel, the increasing prevalence of metabolic disorders has strengthened the need for simple, accessible, and reproducible tools to characterize insulin resistance and cardiometabolic vulnerability in clinical and preventive settings [2]. The triglyceride–glucose (TyG) index, derived from fasting triglycerides (TG) and fasting glucose, has therefore gained attention as a promising surrogate marker of insulin resistance across different stages of the cardiometabolic continuum [2]. Prospective cohort evidence supports its prognostic relevance: in the Vascular Metabolic CUN cohort (VMCUN cohort) (n = 5014; median follow-up 10 years), individuals in the highest TyG quintile exhibited a substantially increased risk of incident CVD compared with those in the lowest quintile (hazard ratio (HR) 2.32, 95% confidence interval (CI) 1.65–3.26), and the addition of TyG to a Framingham-based model modestly improved discrimination (area under the curve (AUC) 0.708 vs. 0.719; *p* = 0.014) [3]. Complementary evidence from the National Health and Nutrition Examination Survey (NHANES) (nine cycles; median follow-up 9.2 years) indicates that TyG may convey mortality information even among participants with normoglycemia, in whom a one-standard-deviation increase in TyG was associated with higher all-cause and cardiovascular mortality (HR 1.35, 95% CI 1.17–1.56; HR 1.38, 95% CI 1.04–1.84) [4]. Beyond cardiovascular outcomes, TyG has also shown clinically relevant performance in metabolic liver dysfunction, with a meta-analysis of 20 studies including 165,725 participants reporting pooled sensitivity 0.73, specificity 0.67, and AUC 0.75 for diagnosing and predicting metabolic dysfunction-associated fatty liver disease/non-alcoholic fatty liver disease (MAFLD/NAFLD) [5].

Dietary carbohydrate quality appears to be a meaningful modulator of glycolipid metabolism and TyG-related profiles, with potential implications for intermediate cardiometabolic risk markers [6]. In PREDIMED-Plus (Prevención con Dieta Mediterránea–Plus) (n = 5373), improvements in a carbohydrate quality index over 6–12 months were associated with concurrent favorable changes across several cardiometabolic traits, including reductions in TG and TyG-related indices [6]. Intervention evidence also indicates that substituting refined cereals with wholegrain cereal-based diets can improve post-prandial metabolic responses, which is mechanistically relevant for triglyceride-rich lipoprotein handling [7]. In a 12-week parallel-group intervention in individuals with metabolic syndrome, a whole-grain cereal-based diet compared with refined cereals significantly reduced post-prandial insulin and TG responses, with average decreases of 29% and 43%, respectively, and significantly lower post-prandial insulin and TG versus controls at study end (*p* = 0.04 and *p* = 0.05) [7]. Observational data further align with this direction, showing inverse associations between whole-grain intake and hypertriglyceridemia, alongside positive associations between refined-grain intake and hypertriglyceridemia and metabolic syndrome [8]. However, evidence from double-blind randomized interventions that test pragmatic, product-level substitution of commonly consumed cereal staples—beyond broad dietary pattern advice—remains limited. In particular, data are scarce on whether functional cereal products formulated to lower the glycemic index can improve the TyG index and complementary vascular function markers in adults with mild fasting hypertriglyceridemia.

To address this gap, we adopted a pragmatic enrichment strategy by enrolling adults with fasting TG > 150 mg/dL, a phenotype in which dietary carbohydrate quality may meaningfully influence insulin resistance-related glycolipid dysregulation captured by TyG [2,6,9]. Accordingly, this double-blind crossover trial will compare functional low-glycemic index pasta, pizza, and flatbread with conventional, commercially available wholegrain counterparts, each consumed as a fixed daily substitution within a stabilized diet, with comprehensive evaluation of TyG and complementary fasting and postprandial glycolipid and cardiometabolic biomarkers.

## 2. Methods

### 2.1. Study Design

This is an exploratory, randomized, double-blind, controlled, single-center crossover trial enrolling healthy adults with suboptimal fasting triglyceride levels. The study aims to compare the effects of a Mediterranean-style dietary pattern enriched with functional low-glycemic index (low-GI) carbohydrate staples versus an otherwise comparable regimen based on conventional wholegrain carbohydrate staples on the TyG index and selected metabolic and vascular biomarkers. Each participant will receive both dietary interventions in a randomized sequence, separated by a pre-specified washout period, enabling within-participant comparisons and improving statistical efficiency by minimizing between-subject variability.

**Figure 1:**
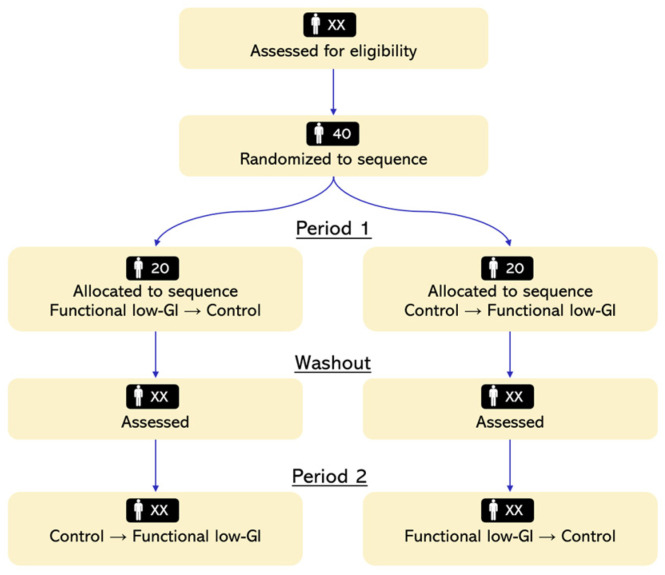
CONSORT-style flow diagram of the GLOW randomized, double-blind crossover trial.

A CONSORT (Consolidated Standards of Reporting Trials)-style flow diagram of participant progression through screening, randomization, crossover periods, washout, and analysis is provided in Figure 1.

### 2.2. Study Setting

The trial will be conducted at the outpatient clinic of the Cardiovascular Medicine Unit, IRCCS Azienda Ospedaliero-Universitaria di Bologna (Policlinico di Sant’Orsola, Bologna, Italy). Biochemical analyses will be performed by trained personnel at the Lipid Laboratory, Department of Medical and Surgical Sciences (DIMEC), Alma Mater Studiorum—University of Bologna.

### 2.3. Eligibility Criteria

Eligibility will be assessed at screening/baseline based on medical history, concomitant therapies, and laboratory findings. Inclusion and exclusion criteria are detailed in Table 1.

The BMI upper limit (≤30 Kg/m^2^) was selected to reduce metabolic heterogeneity and minimize confounding from obesity-related insulin resistance, inflammation, and endothelial dysfunction, thereby enabling a clearer evaluation of the effect of carbohydrate-quality substitution.

### 2.4. Recruitment and Informed Consent

Potential participants will be recruited among adults evaluated at the outpatient clinic. To facilitate enrolment, the study may also be promoted through the sponsoring institution’s official social media channels and, where appropriate, through the Principal Investigator’s professional accounts, in compliance with institutional policies and applicable regulatory requirements. Written informed consent will be obtained prior to the conduct of any study-specific procedures.

### 2.5. Study Products and Administration

Participants will receive two sets of wheat-based cereal staples (pasta and bread-type products), which represent major contributors to habitual carbohydrate intake and are therefore suitable for a pragmatic daily substitution within a Mediterranean-style diet. This product-based substitution strategy was chosen to maximize feasibility, adherence, and generalizability in real-world dietary settings. To preserve blinding, study products will be supplied in neutral, indistinguishable packaging identified only by a study code, with no brand identifiers. The functional intervention comprises Altograno^®^ flour-based pasta, pizza base, and flatbread. These products were selected because their formulation provides higher fiber and protein and lower available carbohydrate than conventional wholegrain products (Table 2), features expected to be associated with a lower glycemic index and attenuated postprandial excursions. The control intervention comprises conventional, commercially available wholegrain pasta, pizza base, and flatbread made from regular wholegrain wheat flour and without additional functional enrichment (e.g., added fibers, resistant starch, or protein concentrates).

Both product sets will be consumed as a fixed daily substitution within a stabilized diet, replacing the usual staple servings typically consumed (one portion of bread and one portion of pasta or pizza per day), without individual dose adjustment. As summarized in Table 2, the product sets are not macronutrient-matched; therefore, the intervention contrast reflects a pragmatic difference in carbohydrate quality and overall nutritional matrix (including fiber, protein, and labeled carbohydrates), rather than glycemic index alone. Study products will be provided free of charge by Casillo SpA (Corato, Bari, Italy).

#### 2.5.1. Nutritional Composition of Study Carbohydrate Products

The nutritional composition of the functional low-glycemic index (low-GI) carbohydrate products (Altograno^®^ pasta, pizza base, and flatbread) and the corresponding conventional wholegrain control products is summarized in Table 2. Values are reported as declared on product labeling; differences in labeled carbohydrates, fiber, and protein reflect the distinct formulations of the functional vs. conventional wholegrain products and are part of the intended product-level substitution.

#### 2.5.2. Product Handling, Storage, and Accountability

Study foods will be delivered to the site prior to screening of the first participant and packaged into predefined kits for each intervention period (28 single-pack pasta portions, 4 pizzas, and 28 flatbread portions). Products will be stored at room temperature (≤25 °C) in a secure area with restricted access, available only to the hospital pharmacist, investigators, or authorized site staff. Products will be managed in accordance with applicable regulatory standards, and treatment logistics will be monitored regularly by the Principal Investigator.

Study products will be pre-numbered, and dispensing will occur only to enrolled participants according to the randomization sequence. The identification number of each dispensed product will be recorded in source documentation and in the electronic case report form (eCRF) using Research Electronic Data Capture (REDCap) to ensure full traceability. Participants will return unused study products and empty or partially used packaging at each visit to support accountability and adherence assessment. Study foods will be used exclusively within the trial.

### 2.6. Randomization, Allocation Concealment, and Blinding

Participants will be randomized in a 1:1 ratio to one of two intervention sequences using computer-generated randomization codes. Randomization codes will be retained in a sealed envelope and accessed only after database lock and completion of the primary data analysis. Study products will be pre-numbered, and dispensing will be documented in source records and in the case report forms.

The study will adopt a double-blind design, with both participants and investigators blinded to treatment allocation. Intervention and control products will be provided in identical packaging and labeled with a study code only. The treatment code will be disclosed only after database lock, unless unblinding is required for safety reasons. Emergency unblinding will be permitted only when deemed essential for clinical management or participant safety and welfare.

### 2.7. Schedule of Assessments

The timing of enrolment, allocation, intervention administration, and study assessments is summarized in Table 3, in accordance with the Standard Protocol Items: Recommendations for Interventional Trials (SPIRIT) guidance. All study parameters will be assessed using standardized methodologies to ensure consistency and reliability of the collected data.

### 2.8. Outcomes

Study outcomes are designed to quantify the comparative effects of functional low-GI foods versus conventional wholegrain foods on glycolipid metabolism and selected vascular biomarkers within a randomized crossover design. Endpoints will be assessed at prespecified trial visits, and analyses will account for treatment, period, and within-participant effects, in accordance with the statistical approach outlined in the protocol.

#### 2.8.1. Primary Endpoint

The primary endpoint is the between-intervention difference in the TyG index response over each 28-day intervention period, defined as the within-participant, period-specific change from the corresponding period baseline (Day 0 for Period 1; Day 56 for Period 2) to the end-of-period assessment (Day 28 and Day 84, respectively). In the primary mixed-effects model, end-of-period TyG will be compared between interventions while adjusting for the corresponding period baseline value, yielding an estimate equivalent to a between-intervention difference in change. The crossover design will enable estimation of within-participant treatment effects while accounting for potential period-related influences.

#### 2.8.2. Secondary Endpoints

Secondary endpoints include between-intervention differences in fasting TG, fasting plasma glucose, non–high-density lipoprotein cholesterol (non-HDL-C), apolipoprotein B (ApoB), and high-sensitivity C-reactive protein (hsCRP). Endothelial reactivity, assessed using the Endocheck^®^/Vicorder^®^ system, is also included among secondary outcomes. In addition, product acceptability will be evaluated at the end of each intervention period using a dedicated questionnaire based on 10-point rating scales assessing overall liking and key sensory attributes (e.g., taste, texture, and appearance). This assessment is intended to capture the tolerability and perceived palatability of the products, which are key determinants of adherence and feasibility in food-based interventions and help support the translational relevance of the study.

#### 2.8.3. Safety Endpoints

Safety endpoints will include the systematic collection and classification of adverse events (AEs), irrespective of their suspected relationship to the study products. Safety monitoring will be conducted throughout the study, and any clinically relevant changes in vital signs or laboratory parameters will be summarized descriptively by intervention period.

### 2.9. Assessments

#### 2.9.1. Laboratory Procedures

At Day −28 and at each subsequent visit, fasting blood samples will be collected to measure fasting plasma glucose, TG, total cholesterol, HDL-C, non-HDL-C, low-density lipoprotein cholesterol (LDL-C), very-low-density lipoprotein cholesterol (VLDL-C), apolipoprotein B100 (ApoB100), apolipoprotein AI (ApoAI), liver enzymes (aspartate aminotransferase (GOT), alanine aminotransferase (GPT), and gamma-glutamyl transferase (gamma-GT)), creatinine, and uric acid [10,11]. All assessments requiring fasting blood sampling will be scheduled in the morning following an overnight fast of at least 12 h; participants will be instructed not to consume any food or caloric beverages (including the study products) before the visit (water allowed). Whenever feasible, visits will be planned at a similar time of day across study periods to minimize diurnal variability. The TyG index will be calculated using the formula ln[fasting TG (mg/dL) × fasting glucose (mg/dL)/2] [9]. Samples will be processed according to standardized laboratory procedures: plasma will be separated by centrifugation shortly after collection, aliquoted into pre-labeled cryovials using coded identifiers to minimize freeze–thaw cycles, and stored in monitored −80 °C freezers with restricted access. Batched measurements will be prioritized when applicable to reduce inter-assay variability. Future ancillary analyses will be limited to cardiometabolic and inflammatory biomarkers consistent with the study objectives and will be conducted in accordance with ethics approval and applicable data protection regulations.

#### 2.9.2. Endothelial Function

Endothelial function will be assessed using the Endocheck^®^ system (BC Biomedical Laboratories Ltd., Vancouver, BC, Canada), integrated within the Vicorder^®^ device, under standardized testing conditions [12]. This cuff-based and largely automated approach provides a standardized assessment of brachial vasoreactivity during reactive hyperemia and has been reported to show acceptable repeatability and reproducibility in methodological evaluations. All assessments will be performed by the same trained operator to minimize inter-operator variability; the operator will undergo initial training and periodic retraining according to the manufacturer’s instructions and site standard operating procedures, consistent with our group’s extensive experience with this methodology in double-blind clinical trials [13,14]. The assessment will be performed during the same morning visit, in the fasting state and before any food intake. Participants will be instructed to remain in the supine position and to refrain from smoking and consuming caffeinated beverages for at least 12 h prior to the assessment [15]. Following a rest period, brachial pulse volume waveforms will be recorded at baseline and during reactive hyperemia induced by 5 min of cuff occlusion. As per the manufacturer’s instructions, one measurement will be obtained at each visit; repeat acquisition will be performed only if required due to inadequate signal quality or artifacts, and the single valid recording will be used for the analyses. Endothelial reactivity will be calculated as √(PV2/PV1), where PV1 and PV2 represent the pulse volume waveform area measured before and during hyperemia, respectively [16].

### 2.10. Adherence and Concomitant Therapies

Participants will be instructed to replace their usual carbohydrate staples with the assigned study products while maintaining an overall carbohydrate intake consistent with their habitual diet. Diet stabilization will be reinforced through scheduled nutritional visits and dietary counseling at each study visit (Table 3), during which any relevant intercurrent dietary changes will be actively reviewed and documented in the electronic case report form (eCRF). Concomitant medications and non-pharmacological products (including dietary supplements and homeopathic preparations) will be recorded in the eCRF. Chronic treatments will be permitted provided they have been stable for at least 3 months prior to enrolment and remain unchanged throughout the study. Adherence will be assessed at each visit using a standard food frequency questionnaire administered as part of the nutritional visit to capture overall dietary patterns and potential compensatory changes in staple carbohydrate intake, and further supported by the return of unused study foods and packaging.

### 2.11. Sample Size Determination

This is a pilot study exploring a comparison that has not been previously tested. The SD assumption (0.7 TyG units) was informed by large population datasets reporting a TyG SD of approximately 0.7 in adults. Assuming a two-sided alpha (α) level of 0.05, 80% power, and an estimated standard deviation (SD) of 0.7 units for the primary endpoint, the required sample size is estimated at 33 participants. With the planned enrolment target of 40 participants, the study will be able to detect a between-intervention difference of approximately 0.30–0.35 TyG units (within-participant, period-specific change), which we considered a clinically meaningful effect size for a proof-of-concept trial in this phenotype. To account for potential dropout (approximately 15%) and post hoc exclusions due to non-compliance, the target sample size will be increased to 40 participants. The study is not powered for secondary endpoints, which will be analyzed as exploratory outcomes and interpreted primarily using effect sizes and 95% confidence intervals to inform future trials.

### 2.12. Statistical Analysis Plan

Analyses will be performed using IBM SPSS Statistics (version 28.0). Continuous variables will be summarized as mean (SD) or median (interquartile range (IQR)), as appropriate, and categorical variables as counts and percentages. Treatment effects will be estimated within the crossover framework using linear mixed-effects models, including treatment, period, and sequence as fixed effects, and participant (nested within sequence) as a random effect to account for within-participant correlation. Accordingly, for the primary endpoint, the treatment effect will be interpreted as the between-intervention difference in period-specific change in TyG. Potential carryover effects will be explored by adding a treatment-by-period interaction term and by comparing period-specific baseline values; if evidence of carryover is observed, sensitivity analyses will be conducted, including analyses restricted to first-period data. Model assumptions (normality of residuals and homoscedasticity) will be assessed; when violated, data transformation and/or non-parametric sensitivity analyses (e.g., Wilcoxon signed-rank test for paired comparisons) will be considered, with paired estimates reported as supportive analyses. A per-protocol analysis will be performed excluding participants with major protocol deviations. Extreme values will be evaluated using the pre-specified approach, including Dixon’s Q test where applicable. All tests will be two-sided, with α = 0.05.

### 2.13. Data Management and Confidentiality

All data will be recorded in study-specific paper and electronic case report forms (CRFs) approved by the Ethics Board and managed through the Research Electronic Data Capture (REDCap)-Unibo platform. All information will be handled as strictly confidential in accordance with Good Clinical Practice (GCP) standards and applicable data protection regulations [17], including the General Data Protection Regulation (GDPR; EU 2016/679) [18]. Data will be stored securely using coded identifiers, restricted access procedures, and protected systems. Any data security breach will be promptly contained, with appropriate impact assessment, required notifications, and corrective actions implemented to prevent recurrence.

### 2.14. Ethics, Insurance, Funding, and Dissemination

The study will be conducted in accordance with International Council for Harmonization (ICH)-GCP, the Declaration of Helsinki (2013) [19], and applicable European and local regulations. The trial will commence only after ethics committee approval and the required institutional authorizations have been obtained, including approval from the IRCCS General Director, and after confirmation of appropriate study-specific insurance coverage. The study is a non-profit initiative sponsored by the University of Bologna (Department of Medical and Surgical Sciences (DIMEC)) and supported by funds allocated to the Principal Investigator under the National Recovery and Resilience Plan (NRRP) “ON Foods” project framework. Results will be disseminated through scientific meetings and peer-reviewed publications, irrespective of study outcomes.

## 3. Discussion

This exploratory, randomized, double-blind, controlled crossover trial is designed to test whether substituting commonly consumed cereal-based staples with functional low-glycemic index alternatives can produce measurable short-term improvements in an integrated marker of insulin resistance-related glycolipid dysregulation, the TyG index, in adults with fasting TG >150 mg/dL. By combining a pragmatic dietary substitution model with a within-participant comparison framework, the study aims to generate proof-of-concept evidence on the metabolic responsiveness of a clinically relevant phenotype characterized by suboptimal triglyceridemia but otherwise preserved health status.

A key strength of the protocol is the selection of TyG as the primary endpoint, given its simplicity, accessibility, and clinical relevance as a surrogate marker of insulin resistance in preventive cardiometabolic medicine [2]. Prospective cohort data support the prognostic value of TyG for incident cardiovascular disease and mortality outcomes, reinforcing its suitability as a clinically meaningful surrogate endpoint in dietary intervention studies [3,4]. In addition, TyG has been associated with vascular aging phenotypes, including arterial stiffness, and with long-term adverse cardiometabolic outcomes, such as incident diabetes, coronary events, stroke, and cardiovascular mortality, further supporting its translational relevance beyond metabolic risk stratification alone [20]. Notably, evidence comparing insulin resistance surrogates suggests that TyG may be a better predictor of cardiovascular risk than the homeostasis model assessment of insulin resistance (HOMA-IR), strengthening the rationale for prioritizing TyG as a primary outcome in cardiometabolic prevention research [21].

The intervention is intentionally designed as a pragmatic “food substitution” strategy, whereby participants replace habitual pasta- and bread-based staples with predefined study products within a stabilized dietary background, thus aiming to minimize behavioral burden and improve real-world applicability [6]. This design choice is relevant because carbohydrate quality—rather than total carbohydrate intake alone—appears to influence intermediate cardiometabolic traits, including TG and TyG-related profiles [6]. Consistent with this concept, intervention evidence in individuals with metabolic syndrome has shown that a whole-grain cereal-based diet (compared with refined cereals) reduces post-prandial insulin and triglyceride responses over 12 weeks, supporting the biological plausibility that cereal quality can modulate dynamic glyco-lipid handling [7]. Furthermore, dietary patterns characterized by adverse glyco-lipid signatures have been associated with higher odds of type 2 diabetes, with TyG contributing as an intermediary marker in the pathway linking dietary exposures and dysglycemia-related outcomes. Together, these observations reinforce the rationale for targeting diet-induced glycolipid dysregulation through feasible, product-level dietary modifications [22].

Mechanistically, the expected direction of effect is likely mediated predominantly through post-prandial pathways, as attenuating glycemic excursions can influence insulin dynamics and downstream hepatic lipid handling, with potential implications for triglyceride-rich lipoprotein metabolism [7]. This is particularly relevant in participants with fasting TG >150 mg/dL, a phenotype in which exaggerated post-prandial lipemia and impaired metabolic flexibility may coexist even in the absence of overt disease [6]. Experimental data also supports a link between refined carbohydrate exposure and early metabolic impairment accompanied by endothelial alterations, including increases in TyG and reduced vascular reactivity in preclinical models, providing additional mechanistic coherence to the hypothesis that carbohydrate quality may influence both metabolic and vascular physiology [23].

In this context, the inclusion of endothelial function assessment provides an additional translational layer by integrating metabolic endpoints with vascular responsiveness [20]. Although the present protocol assesses endothelial reactivity rather than arterial stiffness directly, convergent human evidence supports diet–vascular relationships along the same cardiometabolic axis, including observations that greater adherence to a Mediterranean dietary pattern is associated with lower vascular stiffness parameters across broad community-based samples stratified by insulin resistance status defined through TyG [24]. This alignment supports the clinical plausibility that metabolic improvements captured by TyG may coincide with more favorable vascular function metrics, even over relatively short intervention windows [24].

Beyond the primary endpoint, the secondary biomarker panel (including non–high-density lipoprotein cholesterol and apolipoprotein B) is clinically informative because it may clarify whether any observed metabolic changes translate into broader improvements in atherogenic lipoprotein burden [3]. This is particularly relevant given that TyG-related indices have been linked to more advanced coronary phenotypes when combined with anthropometric parameters, and diet-related insulin load constructs have been associated with greater coronary artery disease severity in at-risk clinical cohorts, suggesting an overarching pathway connecting diet, insulin resistance surrogates, and vascular disease progression [25]. In addition, measuring hsCRP may help contextualize metabolic findings within systemic inflammatory status, which often co-varies with insulin resistance surrogates and cardiometabolic risk profiles [21].

Several methodological strengths enhance the internal validity of this protocol. The randomized crossover design enables within-participant comparisons, improving efficiency and reducing confounding by stable inter-individual characteristics, which is particularly valuable in a pilot setting [6]. The double-blind approach further reduces expectancy effects and performance bias, which can be challenging to control in food-based interventions [7]. Product accountability and structured adherence assessment add robustness by supporting exposure standardization and interpretability of treatment contrasts [6].

Nevertheless, several limitations should be considered. As a single-center pilot trial, findings will primarily inform feasibility, directionality, and effect-size estimation rather than definitive clinical efficacy. Each intervention period lasts 28 days and the protocol relies on surrogate metabolic and vascular biomarkers rather than hard clinical outcomes; accordingly, any observed improvements should be interpreted as short-term biomarker modulation and proof-of-concept evidence, not as durable cardiometabolic risk reduction. Carryover and period effects remain intrinsic to crossover designs, and the planned evaluation of carryover signals and sensitivity analyses restricted to first-period data will be important for preserving interpretability if such effects emerge. Blinding may be challenged by sensory differences between products, and residual variability in background diet may persist despite counseling and stabilization measures. Endothelial reactivity will be assessed using the Endocheck^®^/Vicorder^®^ system; while practical and standardizable for a double-blind intervention setting, this approach is less extensively validated than ultrasound-based FMD and does not have a well-established minimal clinically important difference; therefore, vascular findings will be interpreted primarily using effect sizes and confidence intervals. Moreover, eligibility is restricted to adults with BMI ≤ 30 Kg/m^2^; thus, generalizability to individuals with obesity or more advanced metabolic impairment may be limited. Because the functional and control products differ in fiber, protein, and labeled carbohydrate content, any observed effects will reflect the combined influence of the overall nutritional matrix and carbohydrate quality, and cannot be attributed to glycemic index alone.

If the trial demonstrates favorable changes in TyG and related endpoints, it will provide proof-of-concept support for a scalable “food-as-intervention” strategy in a hypotriglyceridemic phenotype and will inform larger multicenter studies with longer interventions and implementation-oriented outcomes. This may also support more personalized prevention frameworks, as evidence from lifestyle intervention programs suggests that baseline TyG may help stratify individuals who experience more pronounced improvements in TG and glucose-related markers over time [26]. Conversely, if no meaningful differences are observed, the trial will still be informative by refining feasibility parameters, endpoint sensitivity, and the magnitude of any treatment signals, thereby guiding optimization of intervention intensity, duration, and dietary standardization in subsequent studies. Importantly, the broader evidence base supports the clinical relevance of carbohydrate quality, with systematic reviews and meta-analyses reporting modest improvements in insulin resistance-related markers with low-GI dietary patterns and cardiometabolic benefits associated with higher dietary fiber and wholegrain intake [27,28,29,30].

In conclusion, this double-blind crossover trial is designed to provide rigorous, clinically oriented evidence on whether functional low-GI cereal-based staples can favorably modulate the TyG index and complementary cardiometabolic biomarkers in adults with fasting TG >150 mg/dL. By integrating pragmatic dietary substitution with within-participant comparisons and vascular function assessment, the study aims to strengthen the translational foundation for food-based strategies targeting early cardiometabolic risk.

## Figures and Tables

**Table 1:** Eligibility criteria.

Section	Criteria
Inclusion criteria	(1) Male or female aged ≥18 years (2) Fasting TG > 150 mg/dL (3) Ability to communicate and comply with study requirements (4) Written informed consent provided
Exclusion criteria	(1) Gluten hypersensitivity/coeliac disease diagnosis (2) BMI > 30 kg/m^2^ (3) Preventive treatments (e.g., lipid-lowering drugs, antihypertensives) not stabilized in type and dose for ≥3 months (4) Any medical or surgical condition limiting adherence to the protocol

BMI, Body mass index; TG, Triglycerides.

**Table 2:** Nutritional composition of the study carbohydrate products (as provided by product labeling).

Nutrients	Functional Low-GI Products (Altograno^®^ Flour)	Control Products (Standard Wholegrain Flour)
Energy (kJ/kcal)	1413/335	1481/350
Fat (g)	2.5	2.0
Saturates (g)	0.7	0.4
Carbohydrates (g)	53	66
Sugars (g)	4.1	3.0
Fiber (g)	12.0	8.0
Protein (g)	19	13
Salt (g)	0	<0.01

Low-GI, low glycemic index.

**Table 3:** SPIRIT Schedule of Enrolment, Interventions, and Assessments for the GLOW Trial.

Procedures/Assessments	Enrolment (Day −28)	Allocation/Baseline (Day 0)	End of Period 1 (Day 28)	Start of Period 2 (Day 56)	End of Study (Day 84)
Informed consent	X	X			
Eligibility assessment	X	X			
Medical history	X				
Randomization/allocation		X			
Nutritional visit + dietary counseling	X	X	X	X	X
Anthropometrics	X	X			
Hemodynamic tests (BP, HR)	X	X	X	X	X
Blood chemistry (fasting)	X	X	X	X	X
Endothelial function		X	X	X	X
Study food dispense		X		X	
Study food intake (daily)		X	X		
Return of uneaten food			X	X	X
Washout (no study foods)			X	X	
Acceptability evaluation			X		X
Adverse events assessment	X	X	X	X	X

BP, blood pressure; HR, heart rate.

## Data Availability

No data were generated or analyzed for this study protocol. Data generated during the study will not be publicly available due to privacy and ethical restrictions but may be made available from the corresponding author upon reasonable request, subject to ethics approval and applicable data protection regulations (GDPR) and institutional policies.
